# Sound symbolism in manual and vocal responses: phoneme-response interactions associated with grasping as well as vertical and size dimensions of keypresses

**DOI:** 10.1007/s10339-024-01188-y

**Published:** 2024-04-12

**Authors:** L. Vainio, I. L. Myllylä, M. Vainio

**Affiliations:** 1 Perception, Action and Cognition Research Group, Department of Psychology and Logopedics, Faculty of Medicine, University of Helsinki, Haartmaninkatu 3, Helsinki, Finland; 2 Phonetics and Speech Synthesis Research Group, Department of Digital Humanities, University of Helsinki, Unioninkatu 38, Helsinki, Finland

**Keywords:** Sound symbolism, Speech, Manual responses, Articulation

## Abstract

It has been shown that reading the vowel [i] and consonant [t] facilitates precision grip responses, while [ɑ] and [k] are associated with faster power grip responses. A similar effect has been observed when participants perform responses with small or large response keys. The present study investigated whether the vowels and consonants could produce different effects with the grip responses and keypresses when the speech units are read aloud (Experiment 1) or silently (Experiment 2). As a second objective, the study investigated whether the recently observed effect, in which the upper position of a visual stimulus is associated with faster vocalizations of the high vowel and the lower position is associated with the low vowel, can be observed in manual responses linking, for example, the [i] with responses of the upper key and [ɑ] with lower responses. Firstly, the study showed that when the consonants are overtly articulated, the interaction effect can be observed only with the grip responses, while the vowel production was shown to systematically influence small/large keypresses, as well as precision/power grip responses. Secondly, the vowel [i] and consonant [t] were associated with the upper responses, while [ɑ] and [k] were associated with the lower responses, particularly in the overt articulation task. The paper delves into the potential sound-symbolic implications of these phonetic elements, suggesting that their acoustic and articulatory characteristics might implicitly align them with specific response magnitudes, vertical positions, and grip types.

## Introduction

Previous research has shown an interaction between vocalizing particular vowels and consonants and executing precision or power grip responses (Vainio et al. 2013). This interaction effect is here called a grip-sound effect. In the experimental task used by Vainio et al. (2013), participants who were native speakers of Finnish held the precision and power grip device in their right hand, while they were presented with a single vowel (e.g., < i > or < a >) or syllable (e.g., < te > or < ke >) in green or blue color. Their task was to vocalize the visually presented speech unit and simultaneously perform either a precision or a power grip response according to the color of the speech unit. The study revealed the interaction between a vocalized speech unit (e.g., [i]) and the produced grip type (e.g., precision grip), which was observed in facilitated reaction times (RTs) of vocal and manual responses when the vocalized speech unit was congruent with the grip type. In this sound-grip effect, first, the alveolar consonant [t] and the high-front vowel [i] were associated with precision grip responses (i.e., pinching a small object between the tips of the index finger and thumb). Second, the dorsal consonant [k] and the low-back vowel [ɑ] were associated with power grip responses (i.e., clamping a larger object between partly flexed fingers and the palm). More recently, it has been shown that also the high-front vowel [y] and the alveolar consonants [d], [s], and [r] are associated with precision grip responses, while low and high-back vowels [u], [o], and [æ], as well as consonants [l] and [m], whose articulation involves the lowering of the tongue body, are associated with power grip responses (Vainio et al. 2018; Vainio and Vainio 2022).

The grip-sound effects were proposed to reveal a sensorimotor overlap between representations of particular articulatory and grasp gestures (Vainio et al. 2018). As such, this explanation concurred with the accounts assuming tight neural connections between mouth and hand actions (e.g., Corballis 2003; Gentilucci and Campione 2011). It was suggested that this overlap is based on analogies in the goal shape of particular articulatory and grasp actions (i.e., a grasp-articulation hypothesis). For example, the consonant [t] is articulated by moving the tongue tip into contact with the opposing surface of the alveolar ridge and the back of the upper-central incisors forming a narrow pincer shape in the front of the oral cavity. It was speculated that the precision grip, which is produced by bringing the tips of the thumb and index finger into contact, provides a manual counterpart to this articulatory gesture. It was assumed that responding is facilitated when these partially overlapping actions are performed simultaneously in comparison to the conditions in which the two action representations do not overlap.

A recent investigation has, however, challenged the grasp-articulation hypothesis of the grip-sound effect. Heurley et al. (2023) showed a similar effect between manual responses and speech units–which is called here the key-sound effect—when the grasp devices were replaced by small and large response keys. This interaction effect was proposed to show that the grip-sound effect can be based on the compatibility between size codes associated with speech units and responses (the size coding hypothesis) instead of solely compatibility between a particular speech unit and grip type. It was proposed that the precision grip response could be coded as small, and the power grip response could be coded as large as these grip types are typically recruited in grasping small and large objects, respectively. Correspondingly, the speech units [i] and [t] could be coded as small, and the speech units [ɑ] and [k] could be coded as large for one reason or another. When participants have to read one of these speech units and simultaneously perform either the precision or power grip response, responses are facilitated if there is a compatibility between the size codes of the response and the speech unit. Although Heurley et al. did not provide a conclusive proposal for why these speech units would be coded as small/large, below we offer one potential explanation for this coding.

One interesting question that the above-discussed investigations bring up is why these speech units are associated with small/precision and large/power responses. One view that has been previously proposed (Vainio et al. 2019) is that these effects are based–to some extent–on the same cognitive mechanisms as the sound-magnitude symbolism phenomena in which some speech sounds (e.g., [i] and [t]) are associated with small concepts, while some other speech sounds (e.g., [ɑ] and [k]) are associated with large concepts (Newman 1933; Sapir 1929; Taylor and Taylor 1962; Winter and Perlman 2021). The size codes linked to these sound-magnitude symbolism phenomena might be based on the manner in which they are articulated (e.g., the oral cavity is smaller for the [i] than for the [ɑ]) (Ramachandran and Hubbard 2001; Sapir 1929) and/or their specific acoustic properties (e.g., the intrinsic vowel pitch is higher for [i] than [ɑ]) (Ohala and Eukel 1976). Nevertheless, if applying these sound-magnitude symbolism phenomena to explain the grip-sound effect in light of the size coding hypothesis, one might assume that particular speech sounds are abstracted into small/large size codes in a sound-symbolic manner. The manual responses that are targeted to small/large response devices might be also implicitly mapped to these size codes resulting in relatively fast responses when there is a compatibility between the response size and the abstracted size of the speech sound. In contrast, the grasp-articulation hypothesis emphasizes that this mapping between speech sounds and manual responses is rather based on a more concrete representational overlap between a grip type (e.g., pinch with a hand) and articulatory gesture (e.g., pinch with articulators), which contains less cognitive abstraction in mapping the speech unit to the manual response.

Based on the previous observations, it has been proposed that the grip-sound effect might be based on somewhat different processes in relation to producing vowels and consonants. In one study (Vainio et al. 2017), the participants were presented with a picture of a hand shaped to the precision or power grip. They were required to produce the speech sound (e.g., [i] or [ɑ]) according to the front/above perspective of the hand. It was found that the viewed grip type facilitated vocalization responses in the conditions in which the participants had to produce the vowel [i], [ɑ], or [o]. The precision grip facilitated the production of [i] responses, while the power grip facilitated the production of [ɑ] and [o] responses. In contrast, when the participants had to vocalize either [t] or [k], the effect was missing. Contrary to the standard grip-sound effect, the precision grip was not associated with the consonant [t], and the power grip was not associated with the consonant [k]. In another study, the participants were presented with a picture of an object affording either the precision grip (e.g., a pin) or the power grip (e.g., a banana). They were required to produce the speech sound (e.g., [i] or [ɑ]) based on whether the object was natural or man-made. Again, it was found that contrary to the standard grip-sound effect, the objects with the precision/power grip affordance connotations only facilitated the production of [i]/[ɑ] responses, respectively, while the production of [t] or [k] responses was not influenced by perceptual processing of these stimuli.

When explaining these differing grip-sound effects between vowels and consonants, it has been emphasized that decreasing/increasing mouth opening for producing [i]/[ɑ] might provide relatively unambiguous proprioceptive and visual feedback about the magnitude associating these vowels in a relatively abstract manner with conceptual grasp representations (Vainio et al. 2019). In contrast, the articulation of [t] and [k] does not necessarily provide similarly abstract feedback about the magnitude. Rather the grip-sound effect linked to producing these consonants might be grounded in a more concrete mapping between a grip type and an articulatory gesture, which is based on a similar goal shape of articulatory and manual gesture. Therefore, it was proposed that the influence of grasp-related information on the production of the consonants [t] and [k] might require that the grasp is actually performed by a participant themself by, for example, squeezing an object with the precision or power grip (Vainio et al. 2017). That is to say that the vowel-related grip-sound effect might be based on more abstract response coding processes than the consonant-based grip-sound effect. As a consequence, it is possible that the size coding hypothesis only applies to the vowel-related grip-sound effect. If this were the case, one might assume that when the grasp devices were replaced by small and large response keys, the effect can be observed with the speech units of [i] and [ɑ] but not with the speech units of [t] and [k]. However, this hypothesis has not yet been explored as Heurley et al. (2023) asked their participants to read syllables [ti] and [kɑ] that both contain vowel and consonant elements that are hypothetically congruent with a particular response size. Hence, the primary objective of the present study was to investigate whether the effect between speech sounds and manual responses could be similarly observed when the participants were required to read either the vowels [i] or [ɑ], or the consonants [t] or [k].

Experiment 1 replicated the original grip-sound paradigm reported by Vainio et al. (2013) with the exception that instead of using different grip types for manual responses, in Experiment 1a, participants were required to produce responses with the small and large response keys, while in Experiment 1b, manual responses were performed with the precision and power grip device as in the original study. In Experiment 2, we aimed to further test whether the response-sound effect differs between large/small key presses and precision/power grasps by requiring participants to read these speech units silently while producing these manual responses, similarly to the paradigm reported by Vainio et al. (2014). Again, in Experiment 2a, the manual responses were performed with the small and large response keys whereas in Experiment 2b, the manual responses were performed with the precision and power grip device. Experiment 2 was carried out to investigate whether overt articulation of a speech unit (Experiment 1) produces different grip-sound or key-sound effects than reading speech units without overt articulation (Experiment 2). This distinction in the overt and covert reading of the speech units between experiments presents another main objective for the study. It is hypothesized that if the effect is based on articulatory movements or acoustic consequences of these movements, the effect should be greater in the condition of overt articulation. Finally, similarly to the original studies, the investigations of the present study only recruit native speakers of Finnish.

Although the effects are primarily expected to operate in the manual and vocal reaction times, the study also explores whether these effects can manifest themselves in the acoustic vocal characteristics of intensity, fundamental frequency (*f*_*0*_), first formant (*F*_*1*_), and/or second formant (*F*_*2*_). It is known that high-front vowels have typically slightly higher *f*_*0*_ than low and back vowels (Whalen and Levitt 1995). This phenomenon is known as the intrinsic vowel pitch. Given that small things and animals are typically associated with a higher pitch than large things and animals (Ohala 1995), we hypothesize that if responding with a small/large response key or precision/power grip device would modulate *f*_*0*_ values, the small/precision responses are expected to heighten these values in comparison to the large/power responses. Furthermore, it is known that the low vowels have typically higher *F*_*1*_* values* than the high vowels, and the front vowels have higher *F*_*2*_ values than the back vowels due to differences in their articulatory configurations (Fant 1960). As such, if manual performance influences articulatory performance in a systematic manner, it might be expected that performing the small/precision responses, in comparison to large/power responses, could facilitate forming a narrow pincer shape into the front of the oral cavity, which in turn could be observed in lowered *F*_*1*_ values and heightened *F*_*2*_ values.

### Interaction between speech units and upper/lower responses

It has been recently presented that the high-front vowel [i] is implicitly associated with the concepts of *up* and *above*, while the low-front vowel [æ] is associated with the concepts of *down* and *below* (Vainio et al. 2023b). In that study, the participants were required to pronounce either [i] or [æ] based on whether a target stimulus moved upward or downward, or depending on whether the target appeared above or below the reference object. It was found that [i] was produced faster in the upward and above conditions, while [æ] was produced faster in the downward and below conditions. It was suggested the same cognitive mechanisms might underlie this effect as also underlies the pitch-elevation effect (Pratt 1930; Shintel et al. 2006; Spence 2019) in which high-pitch sounds were associated with an upper visual position, while low-pitch sounds were associated with a lower visual position. According to this view, the [i] might be linked to the upper visual position and [æ] to the lower position because [i] has a higher intrinsic vowel pitch than [æ] (Whalen and Levitt 1995). Alternatively, given that [i] is produced by moving the tongue to the high position, while [æ] is produced by moving it to the low position, it was proposed that [i]/[æ] might be associated with up/down, respectively, because of the sensorimotor congruence between the given spatial concept and the articulatory movement.

The present study investigated this phenomenon by replacing the visual upper/lower stimuli with the upper/lower responses. Notice that although the response keys are termed as ‘upper’ and ‘lower’, these keys are located so that the responses are performed in the sagittal axis in the same way as in the study of Rusconi et al. (2006). The participants were required to read either the vowels [i] or [ɑ], or the consonants [t] or [k], and simultaneously respond by pressing manually either the upper or lower response key with their right hand according to the color in which the speech unit is presented. As such, the study presents a version of the SMARC (Spatial–Musical Association of Response Codes) paradigm. Originally, the SMARC task was used to investigate the pitch-elevation effect (Rusconi et al. 2006). It showed that the pitch-elevation effect, discussed above, can also manifest itself in manual upper-lower responses. That is, the upper response key is pressed faster when the auditory target stimulus has a higher pitch, while the lower response key is pressed faster when the target stimulus has a lower pitch. Correspondingly, the presented study explores whether the upper response key is pressed faster when the participant is required to read the high-front vowel [i] in comparison to the low-back vowel [ɑ], and whether the lower response key is pressed faster when the participant is required to read [ɑ] in comparison to [i]. Furthermore, the study investigates whether this phenomenon can be also observed with the consonants [t] and [k]. It is expected that if the effect can be observed with these consonants, the consonant [t] would be more likely to be linked to faster responses with the upper key and the [k] would be linked to faster responses with the lower key. This is assumed because the alveolar stop consonant [t] has higher spectral components in the release of the stop than the dorsal stop consonant [k] (Chodroff and Wilson 2014), which could be a factor in making [t] acoustically suitable for the depiction of the upper location. Additionally, [t] could be a better match for the upper location than [k] because when articulating [t], the tongue tip moves upwards, while when articulating [k], the tongue tip moves downwards.

Finally, it should be mentioned that the experimental setup for investigating the interaction between the speech units and upper/lower responses was subordinate to the experimental setup for investigating the interaction between the speech units and small/large responses. That is, the primary goal of these studies was to investigate how reading particular speech units influences responding with small and large response keys/grips, and the research questions related to the upper/lower locations of the response keys were investigated within the experimental frames of this primary research question. Hence, the stimulus (i.e., the visually presented vowels and consonants) were primarily selected for investigating the main research question. However, it is noteworthy that the same speech units work fairly well for investigating the interaction between speech units and upper/lower responses (see the General Discussion for the potential limitations of using these speech units).

### Experiments 1a and 1b

Experiment 1 investigates how vocalizing vowels [i] and [ɑ], or the consonants [t] and [k], influence producing manual responses with the small/large response key (Experiment 1a) or precision/power grip device (Experiment 1b), and how producing these manual responses influences vocalizing these speech units. In addition to testing whether some difference can be observed in the key-sound and grip-sound effects, Experiment 1a also investigates whether vocalizing these same speech units influences producing upper/lower responses and whether performing upper/lower responses influences vocalizing these speech units in some systematic manner.

## Methods

### Participants

Eighteen volunteers naïve to the purposes of the experiment participated in Experiment 1 (20–41 years of age; mean age = 26.5 years; 8 males; 1 left-handed). All participants were native speakers of Finnish and reported normal hearing and normal or corrected-to-normal vision. Statistical power was estimated based on simulations (Brysbaert and Stevens, 2018). The simulations were carried out on an earlier dataset from an experiment with a very similar design (Vainio and Vainio 2022). The simulation script and the dataset (ref_data) are provided in https://osf.io/u3pxe/. In the simulations, a mixed linear model with log-transformed reaction time data was fitted. The participants had a random effect on the intercept and the slope of congruency. The simulations suggest, firstly, that with the effect size (d_z_ = 0.67) observed by Vainio and Vainio (2022–the congruency effect for Experiment 1; Block 1), 16 observers would have sufficed to produce a statistically significant difference in 100% of experiments. The simulations were run with R package simr (Green and MacLeod 2016). Written informed consent was obtained from all participants. The study was conducted according to the principles expressed in the Declaration of Helsinki. The study was approved by the Ethical Review Board in the Humanities and Social and Behavioral Sciences at the University of Helsinki.

### Stimuli, procedure, and apparatus

Each participant sat in a dimly lit room with his or her head 75 cm in front of a 19″ CRT monitor (screen refresh rate: 85 Hz; screen resolution: 1280 × 1024). A head-mounted microphone was adjusted close to the participant’s mouth. At the beginning of each trial, a blank white screen was presented for 2000 ms. Then the grey fixation cross was presented for 400 ms at the center of the screen. The blank screen was again displayed for 500 ms after the offset of the fixation cross. Then the target stimulus was presented for 1500 ms at the location of the fixation cross. The target was a letter < a > (i.e., [ɑ]) (horizontally: 1°; vertically: 1.2°; respectively in centimeters: 1.3 cm/1.6 cm) or < i > (0.9°; 1.5°; 1.2 cm/1.9 cm) [or a syllable < ke > (2.1°; 1.5°; 2.7 cm/2 cm) or < te > (2.2°; 1.5°: 2.9 cm/2 cm) in block 2] presented in green (R = 2, G = 157, B = 14) or blue (R = 34, G = 22, B = 250) color. They were written in lowercase letters with the Consolas font.

**Fig. 1:**
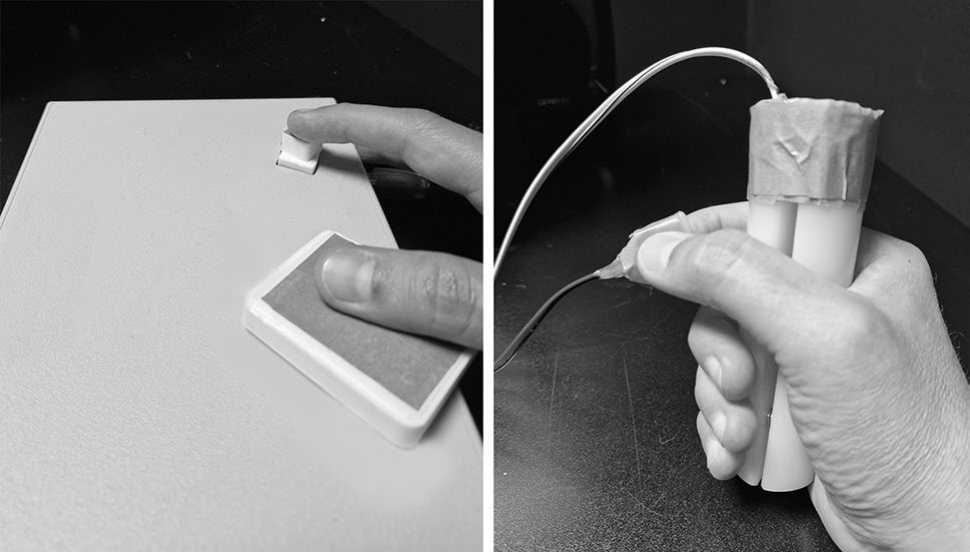
The response devices for the keypress responses (left) and the grip responses (right)

In Experiment 1a, the participants responded by pressing one of the two alternative response keys of the Cedrus response pad (see Fig. 1 left). The response pad was located at the front of the monitor. One key was closer to the monitor (i.e., the upper key) and one key was closer to the participant (i.e., the lower key). The distance between the two keys was 10.2 cm measured between the centers of the two keys. The upper key was pressed with the index finger and the lower key was pressed with the thumb. All participants used their right hand for the responses. One response key was small (1.8 cm × 1.7 cm) and one was large (6.7 cm × 5.0 cm). A blue or green sticker was placed on each response key. Based on the size of the keys, one sticker was large and the other one was small. The only difference between Experiment 1a and 1b was that in 1b the participants responded with grip devices. There were two grip response devices, each equipped with an inlaid micro-switch: the precision grip device (2 cm × 2 cm) and the power grip device (11 cm long, 3.2 cm diameter) (see Fig. 1 right). A blue or green sticker was placed on each grip device. The participants held the grip devices in their right-hand. Each participant carried out Experiment 1a before Experiment 1b. This was because we wanted to avoid any carry-over influence from the grip response task to the keypress task. The effect observed with grip squeeze responses is a much more established finding than the corresponding effect observed with keypresses and—contrary to the finding observed with keypresses—can be evidently observed separately for vowels and consonants even when the grip response task does not follow the keypress task. Furthermore, the absolute objective of the study was to explore how particular speech responses influence exclusively keypress responses. We did not want to risk that the grip block would have any carry-over effect on these observations.

Experiments 1a and 1b consisted of two blocks. The participants had a short break between the blocks. In one block, the stimuli consisted of the vowels < i > and < a > , while the stimuli in the other block consisted of the syllables < te > and < ke > . The order of the blocks was randomized between the participants. In addition, the order of the differing stimuli was randomized. In Experiment 1a, there were four different response settings: 1a) the large-green key upper; the small-blue key lower; 2a) the large-blue key upper; the small-green key lower; 3a) the small-green key upper; the large-blue key lower; 4a) the small-blue key upper; the large-green key lower. Notice that in the analysis of Experiment 1a, the response size and the vertical response location were separated so that in the analysis, which focused on testing potential interaction between the Speech unit and Response size, the response size was treated as an independent within-subjects variable over the vertical response location. In contrast, in the analysis that focused on testing potential interaction between Speech unit and Response location, the response location was treated as an independent within-subjects variable over the response size. The same logic is also applied to analyzing the data of Experiment 2a. From the first sixteen participants who participated in the study, four participants were allocated to each of these response settings. The two final recruits were randomly allocated to these response settings. In Experiment 1b, there were two different response settings: 1b) the precision grip-blue; the power grip-green; 2b) the precision grip-green; the power grip-blue. Half of the participants performed the task in response setting number 1b. There was a break between Experiment 1a and 1b that lasted for 10 min. During that break, the grip task was introduced to the participants.

The participants were asked to press the key (or squeeze the device in Experiment 1b) according to the color of the target as fast and accurately as possible. In addition to making the manual responses, the participants were asked to pronounce the vowel/syllable at the same time as they gave the manual response. It was emphasized that the vowel/syllable should be uttered promptly at the participant’s natural pitch and intensity level. The participants were given enough time to practice until they felt comfortable with the task. This practice phase required on average 18 trials. The experiment was not started until the participant was able to perform accurate and fast manual and vocal responses simultaneously. Estimation of the adequate response speed, accuracy, and simultaneity was based on the experimenter’s observation. In addition, there was a practice session of eight trials at the beginning of each new block in which the participants were familiarized with speech units that had to be pronounced in the upcoming block. Erroneous manual responses were immediately followed by a short “beep” tone in the practice as well as in the actual experiment. In total, Experiments 1a and 1b lasted around 10 min and consisted of 240 trials [30 × 2 (block) × 2 (speech unit) × 2 (response)] with the exception that the experiments consisted of 256 trials (32 × 2 × 2 × 2) for the first two participants. The number of trials was smaller for the sixteen first participants because they had to perform an additional experiment (not reported in the paper) after this one, and therefore we wanted to make the experiment a bit shorter for those participants.

Sound recording and stimulus presentation were carried out with Presentation^®^ software (Version 16.1, www.neurobs.com). The vocal responses were recorded for 2000 ms starting from the onset of the target object. At the beginning of the experiment, the recording levels were calibrated for each participant using the voice calibration function of Presentation^®^ software so that the recording levels would match the natural intensity of the participant’s voice.

### Statistical analyses

The onsets of the vocalizations were located individually for each trial as the first observable peak in the acoustic signal for the vowel and the consonant burst by Praat (Boersma 2001). The offsets of the vocalizations were located individually for each trial as the observable ending of the acoustic signal. The spectral components (*F*_*1*_ and *F*_*2*_), as well as *f*_*0*_, were calculated as median values of the middle third of the voiced section of the vowel. The intensity in decibels (dB) was calculated as the maximum value of the voiced section.

The following parameters were analyzed from the raw data as dependent variables: vocal reaction times (RT), manual RTs, dB, *f*_*0*_, *F*_*1*_, and *F*_*2*_. On a few occasions, the formant value was not found by Praat (Version 6.2.15) or the software mixed an *F*_*1*_ value with an *F*_*2*_ value, for example, due to breathy voice quality as some participants produced vocalizations rather quietly. As an example, six data points of *F*_*1*_ (1234 Hz, 2051 Hz, 2035 Hz, 2085 Hz, 2015 Hz, 2100 Hz) were removed from the [i] vocalizations of participant 5 (grip responses) because after removing these values, the values range between 231 and 386 Hz (sd = 35 Hz). For the data of participant number 15, all *F*_*1*_ values had to be removed for the vowel [ɑ] because these values were not properly detected by Praat. Additionally, on a few occasions, the output value clearly exceeded variations that can normally be observed within the voice characteristics of the given vowels (e.g., octave jump errors). The missing values and outliers were discarded before analyzing the acoustic characteristics of the vocalizations (Experiment 1a: intensity: 0.0%; *f*_*0*_: 3.6%; *F*_*1*_: 3.9%; *F*_*2*_: 0.8%; Experiment 1b: intensity: 0.0%; *f*_*0*_: 3.7%; *F*_*1*_: 3.6%;* F*_*2*_: 1.3%). However, prior to analyzing RTs and any of these vocal parameters, the errors were removed from the data (i.e., the participant produced a wrong manual response: Experiment 1a: 1.6%; Experiment 1b: 0.9%; the participant did not produce any response: Experiment 1a: 0.2%; Experiment 1b: 0.0%). The reaction times below 150 ms (Experiment 1a: vocal: 0.3%, manual: 0.0%; Experiment 1b: vocal: 0.4%, manual: 0.0%) were removed from the data (see Miller 2023 for the outlier exclusion procedures). In addition, for analyzing fundamental frequencies, the raw *f*_*0*_ values were converted to semitones (st) relative to each participant’s mean *f*_*0*_. Semitone conversion was conducted to account for the logarithmic nature of perceiving pitch and pitch movements and to eliminate the bimodal distribution of fundamental frequencies caused by male speakers having fundamentally lower *f*_*0*_ values than female speakers.

To sum up, Experiment 1 consisted of two separate experiments that were named Experiment 1a and 1b. In Experiment 1b, the responses were performed using grip devices and in Experiment 1a the responses were performed using response keys from which one was small and located at either upper or lower vertical location, while one was large and located at either upper or lower vertical location. In addition, the vertical location and key size were treated as independent variables in separate analyses of Experiment 1a. Both Experiments consisted of two blocks from which one required vocalizing the vowels [i] and [ɑ] whereas the other required vocalizing the syllables [te] and [ke]. The data from Experiment 1a was analyzed in two separate analyses. One analysis focused on the response size and included three independent variables with two levels that were Block (1 = vowels, 2 = syllables), Speech unit (1 = [i]/[te], 2 = [ɑ]/[ke]), and Response size (1 = small, 2 = large). The other analysis focused on the vertical location and included three independent variables with two levels that were Block (1 = vowels, 2 = syllables), Speech unit (1 = [i]/[te], 2 = [ɑ]/[ke]), and Response location (1 = upper, 2 = lower). Experiment 1b included three independent variables with two levels that were Block (1 = vowels, 2 = syllables), Speech unit (1 = [i]/[te], 2 = [ɑ]/[ke]), and Grip type (1 = precision, 2 = power).

The statistical significance of observed differences was tested using the generalized linear mixed model (GLMM) analysis framework, which can be used even when the residual normality assumption and the independence of observations assumption do not hold. In the current study, for dependent variables RT, intensity, *F*_*1*_, and *F*_*2*_, the residual distributions were significantly positively skewed, so a gamma distribution assumption (log link function) was implemented. For dependent variable *f*_*0*_ (converted to semitones), the residual distribution was approximately normal, and a normal distribution assumption (identity link function) was implemented. The GLMM analyses treated the independent variables of Block, Speech unit, and Response (see above) as fixed within factors. In all of the reported analyses, Subject was a random intercept, and the independent variables were treated as random slopes. All pairwise comparisons were carried out using Bonferroni correction for multiple comparisons. The analyses were carried out using the SPSS statistics software package (version 28). The output tables of Experiments 1 and 2 are provided in https://osf.io/u3pxe/.

## Results and discussion

### Experiment 1a (keypress)

*The interaction between the speech unit and the small/large response*: The analysis of *vocal* RTs revealed a significant two-way interaction between Speech unit and Response size [F(1,4250) = 33.58, *p* < 0.001]. The three-way interaction between Block, Speech unit, and Response size was also significant [F(1,4250) = 6.80, *p* = 0.009]. The interaction between Speech unit and Response size was significant for both vowels in the vowel block ([i]-small: M = 615 ms vs. [i]-large: M = 639 ms, *p* = 0.005, *d*_*z*_ = 0.19; [ɑ]-small: M = 634 ms vs. [ɑ]-large: 603 ms, *p* < 0.001, *d*_*z*_ = 0.23). However, corresponding interactions were not significant in the consonant block ([te]-small: M = 631 ms vs. [te]-large: M = 641 ms, *p* = 0.235; [ke]-small: M = 636 ms vs. [ke]-large: 626 ms, *p* = 0.209).

**Fig. 2:**
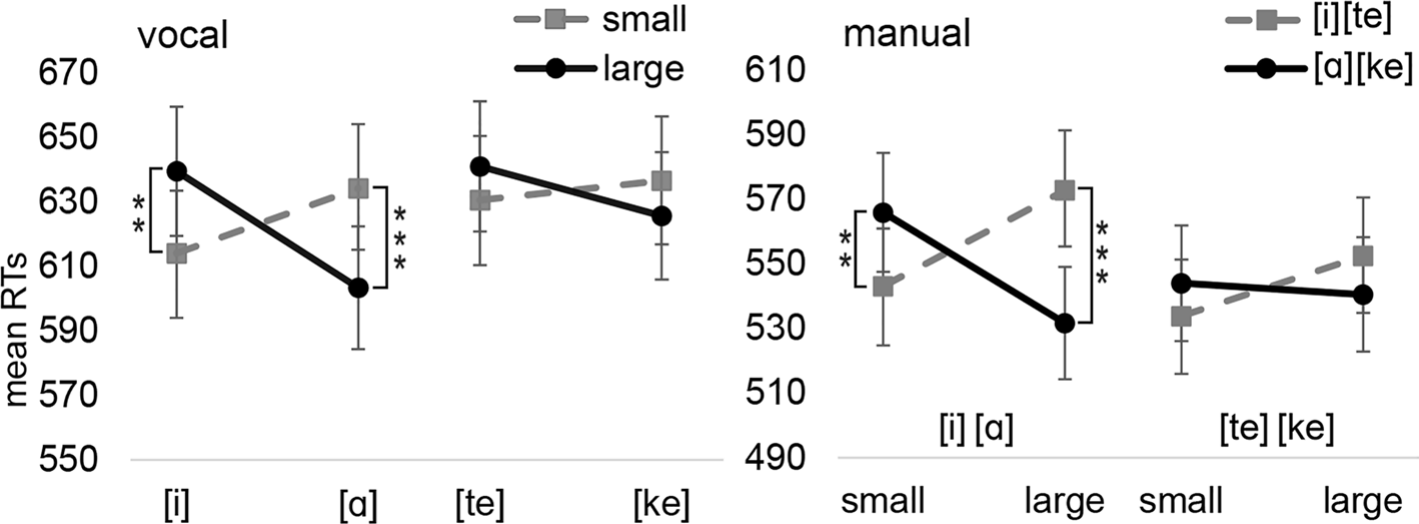
The mean vocal and manual reaction times for Experiment 1a as a function of the speech unit and response size. Error bars depict the standard error of the mean. Asterisks indicate statistically significant differences (*p < .05, **p < .01, ***p < .001)

Similarly to vocal RTs, the analysis on *manual* RTs revealed the interaction between Speech unit and Response size [F(1,4264) = 36.68, *p* < 0.001]. The three-way interaction was also significant [F(1,4264) = 8.17, *p* = 0.004]. Speech unit and Response size showed a significant interaction for both speech units in the vowel block (small-[i]: M = 544 ms vs. small-[ɑ]: M = 566 ms, *p* = 0.007, *d*_*z*_ = 0.20; large-[i]: M = 573 ms vs. large-[ɑ]: M = 532 ms, *p* < 0.001, *d*_*z*_ = 0.37). However, the corresponding interactions were not significant in the consonant block (small-[te]: M = 534 ms vs. small-[ke]: M = 544 ms, *p* = 0.196; large-[te]: M = 552 ms vs. large-[ke]: M = 541 ms, *p* = 0.131). The vocal and manual interactions are depicted in Fig. 2.

We also analyzed the data of *manual* RTs so that the variables of Speech unit and Response size were transformed into a single variable of Congruency [1 = congruent ([i]/[te]-small, [ɑ]/[ke]-large), 2 = incongruent ([i]/[te]-large, [ɑ]/[ke]-small)]. Other variables in this analysis were Response location (1 = upper, 2 = lower) and Block (1 = vowels, 2 = consonants). The analysis tested whether the congruency effect between Speech unit and Response size is significantly different between the vowel and consonant block and whether the location of the response key influences the congruency effect between a speech unit and the small/large size of the response. This analysis revealed an interaction between Block and Congruency [F(1,4264) = 8.23, *p* = 0.004] showing that the congruency effect is significantly larger in the vowel block (congruent: M = 538 ms vs. incongruent: M = 569 ms, *p* < 0.001, *d*_*z*_ = 0.30) than in the consonant block (congruent: M = 537 ms vs. incongruent: M = 548 ms, *p* = 0.165, *d*_*z*_ = 0.09). However, the response location did not influence the congruency effect (Response location*Congruency: *p* = 0.100; Response location*Block*Congruency: *p* = 0.786).

The analysis of intensity in decibels (dB) showed a significant main effect of Block [F(1,4264) = 12.83, *p* < 0.001] (the vowel block: M = 76.5 dB vs. the consonant block: M = 75.4 dB, *d*_*z*_ = 0.22). Concerning *f*_*0*_ values, the main effect of Speech unit [F(1,4112) = 20.86, *p* < 0.001] as well as the interaction between Block and Speech unit [F(1,4112) = 121.58, *p* < 0.001] were significant. In the vowel block, *f*_*0*_ values were higher for [i]-responses (M = 0.26 st) than [ɑ]-responses (M = -0.20 st) (*p* < 0.001, d_z_ = 0.65). In the consonant block, this interaction was not significant ([te]-responses: M = 0.02 st vs. [ke]-responses: M = 0.10 st, *p* = 0.82).

Regarding *F*_*1*_ values, there were significant main effects of Block [F(1,4097) = 10.24, *p* < 0.001] and Speech unit (as can be expected from the different articulatory configurations) [F(1,4097) = 74.82, *p* < 0.001] and a significant interaction between Block and Speech unit [F(1,4097) = 4478.28, *p* < 0.001]. In the vowel block, *F*_*1*_ values were higher for [ɑ]-responses (M = 546 Hz) than [i]-responses (M = 320 Hz) (*p* < 0.001, d_z_ = 1.81). In the consonant block, this interaction was not significant ([te]-responses: M = 461 Hz vs. [ke]-responses: M = 458 Hz, *p* = 0.870). When* F*_*2*_ values were analyzed, there were significant main effects of Block [F(1,4229) = 14.72, *p* < 0.001], Speech unit [F(1,4229) = 2354.71, *p* < 0.001], and a significant interaction between Block and Speech unit [F(1,4229) = 77,118.01, *p* < 0.001]. In the vowel block, *F*_*2*_ values were higher for [i]-responses (M = 2674 Hz) than [ɑ]-responses (M = 1093 Hz) (*p* < 0.001, d_z_ = 4.51). In the consonant block, this interaction was not significant ([te]-responses: M = 1831 Hz vs. [ke]-responses: M = 1849 Hz, *p* = 0.331).

*The interaction between the speech unit and the upper/lower response*: The analysis of *vocal* RTs revealed a significant two-way interaction between Speech unit and Response location [F(1,4250) = 44.34, *p* < 0.001] as well as a significant three-way interaction between Block, Speech unit and Response location [F(1,4250) = 5.18, *p* = 0.023]. The Speech unit * Response location interaction was significant for other speech units ([i]-upper: M = 613 ms vs. [i]-lower: M = 641 ms, *p* < 0.001, d_z_ = 0.21; [ɑ]-upper: M = 636 ms vs. [ɑ]-lower: 601 ms, *p* < 0.001, d_z_ = 0.3; [ke]-upper: M = 646 ms vs. [ke]-lower: 616 ms, *p* < 0.001, d_z_ = 0.19) but [te] ([te]-upper: M = 634 ms vs. [te]-lower: M = 638 ms, *p* = 0.648).

**Fig. 3:**
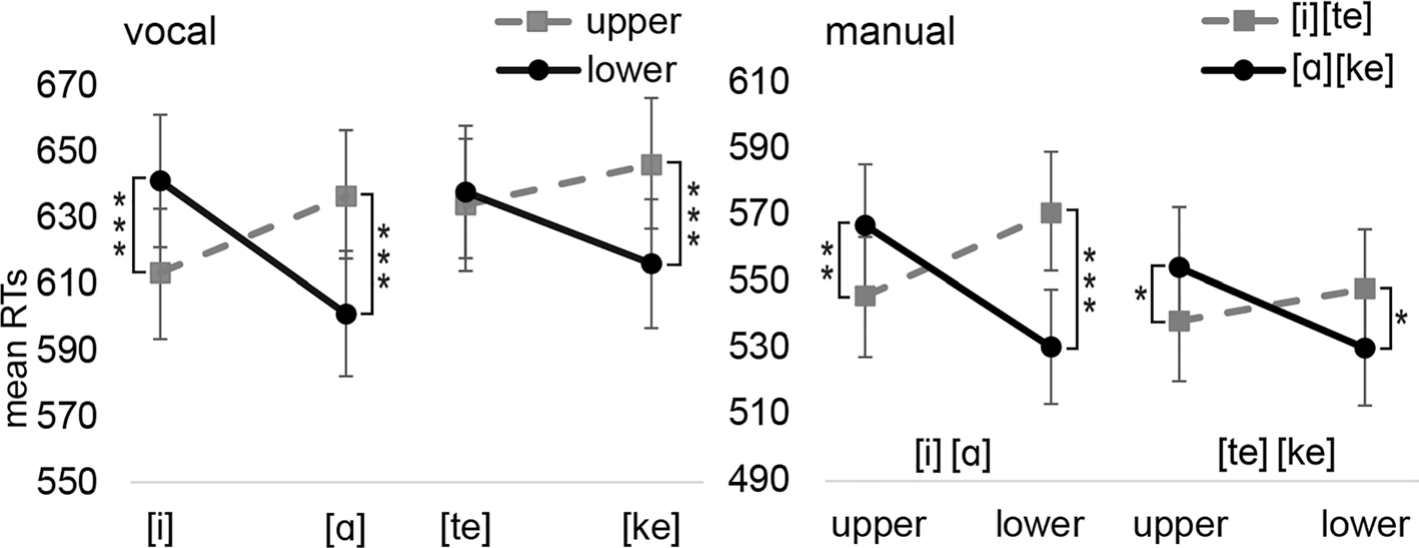
The mean vocal and manual reaction times for Experiment 1a as a function of the speech unit and vertical response location. Error bars depict the standard error of the mean. Asterisks indicate statistically significant differences (*p < .05, **p < .01, ***p < .001)

The analysis of *manual* RTs revealed the same interaction between Speech unit and Response location [F(1,4264) = 46.36, *p* < 0.001]. The three-way interaction was marginally significant [F(1,4264) = 3.62, *p* = 0.057]. The interaction between Speech unit and Response location was significant between Speech units in upper and lower locations (upper-[i]: M = 546 ms vs. upper-[ɑ]: M = 567 ms, *p* = 0.009, d_z_ = 0.19; lower-[i]: M = 571 ms vs. lower-[ɑ]: M = 531 ms, *p* = 0.043, d_z_ = 0.35; upper-[te]: M = 538 ms vs. upper-[ke]: M = 554 ms, *p* = 0.020, d_z_ = 0.15; lower-[te]: M = 548 ms vs. lower-[ke]: M = 530 ms, *p* = 0.023, d_z_ = 0.16). Figure 3 presents the interactions between Speech units and Response locations for vocal and manual responses.

We also analyzed the data of *manual* RTs so that the variables of Speech unit and Response location were transformed into a single variable of Congruency [1 = congruent ([i]/[te]-upper, [ɑ]/[ke]-lower), 2 = incongruent ([i]/[te]-lower, [ɑ]/[ke]-upper)]. Other variables in this analysis were Response size (1 = small, 2 = large) and Block (1 = vowels, 2 = consonants). This analysis was carried out to explore whether Response size influences the congruency effect between Speech unit and Response location and whether this congruency effect differs between the blocks. As already known, the main effect of congruency was significant [F(1,4264) = 12.85, *p* < 0.001]. However, the analysis did not reveal any significant interactions (Block*Congruency: *p* = 0.054; Response size*Congruency: *p* = 0.097; Response size*Block*Congruency: *p* = 0.248) suggesting that the Size or Block does not influence this congruency effect.

The analyses of the vocal characteristics that treated Block, Speech unit, and Response location as independent variables, did not reveal any significant main effects or interactions besides those main effects that are already reported in the context of the analysis, reported above, which explores the interaction between the speech unit and the small/large response (e.g., *f*_*0*_ and *F*_*2*_ are higher for the vowel [i] than the vowel [ɑ]).

### Experiment 1b (grip squeeze)

*The interaction between the speech unit and the precision/power response*: The analysis of *vocal* RTs revealed significant main effects of Block [F(1,4287) = 8.83, *p* = 0.003] (Vowels: M = 597 ms; Consonants: M = 625 ms). The interaction between Speech unit and Grip type was also significant [F(1,4287) = 91.52, *p* < 0.001]. The interaction between Block, Speech unit, and Grip type was not significant (*p* = 0.076). The p-values of the Speech unit*Grip type interaction were significant for both Speech units across blocks with the exception that the interaction was not significant for the interaction between the vowel [ɑ] and the power grip ([i]-precision: M = 583 ms vs. [i]-power: M = 618 ms, *p* < 0.001, d_z_ = 0.26; [ɑ]-precision: M = 598 ms vs. [ɑ]-power: M = 587 ms, *p* = 0.086, d_z_ = 0.09; [te]-precision: M = 611 ms vs. [te]-power: M = 650 ms, *p* < 0.001, d_z_ = 0.28; [ke]-precision: M = 636 ms vs. [ke]-power: M = 603 ms, *p* < 0.001, d_z_ = 0.23).

**Fig. 4:**
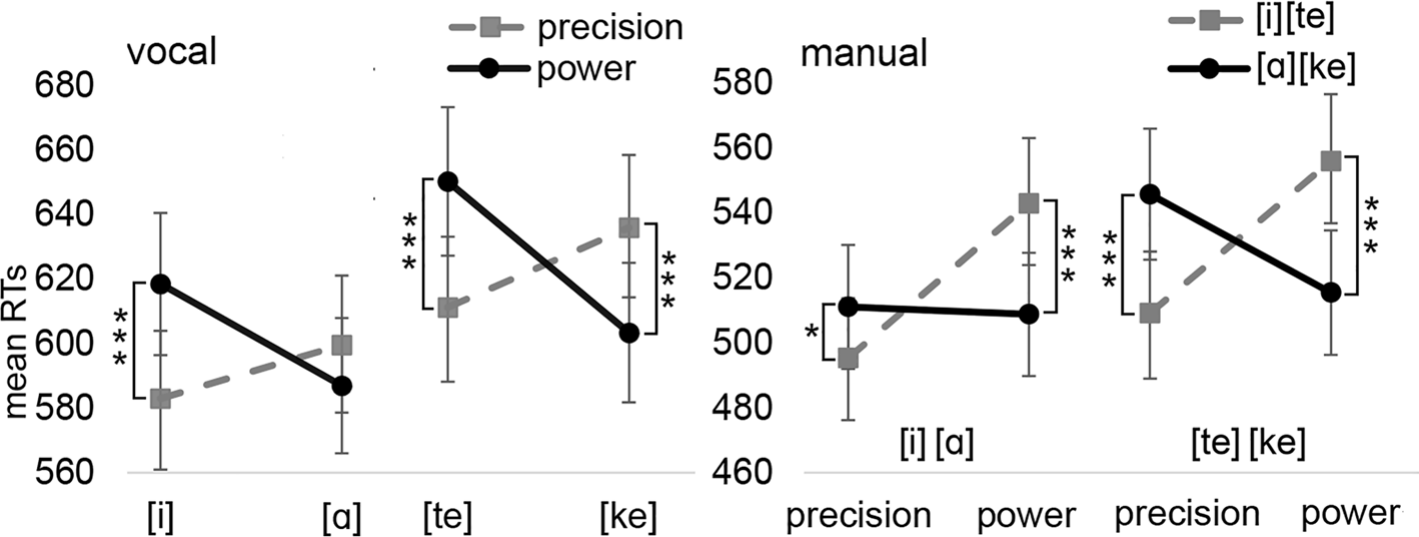
The mean vocal and manual reaction times for Experiment 1b as a function of the speech unit and grip type. Error bars depict the standard error of the mean. Asterisks indicate statistically significant differences (*p < .05, **p < .01, ***p < .001)

The analysis on *manual* RTs revealed a significant main effect of Grip type [F(1,4303) = 6.33, *p* = 0.012] (precision: M = 515 ms vs. power: M = 530 ms, d_z_ = 0.13) as well as the interactions between Speech unit and Grip type [F(1,4267) = 82.17, *p* < 0.001] and Block and Grip type [F(1,4303) = 87.99, *p* < 0.001]. The three-way interaction was not significant (*p* = 0.058). The interaction between Speech unit and Grip type was significant for both speech units in the vowel and consonant blocks (precision-[i]: M = 495 ms vs. precision-[ɑ]: M = 511 ms, p = 0.023, d_z_ = 0.14; power-[i]: M = 543 ms vs. power-[ɑ]: M = 509 ms, *p* < 0.001, d_z_ = 0.28; precision-[te]: M = 509 ms vs. precision-[ke]: M = 546 ms, *p* < 0.001, d_z_ = 0.31; power-[te]: M = 556 ms vs. power-[ke]: M = 515 ms, *p* < 0.001, d_z_ = 0.33). The observed interactions are shown in Fig. 4.

We also analyzed the data of *manual* RTs so that the variables of Speech unit and Grip type were transformed into a single variable of Congruency [1 = congruent ([i]/[te]-precision, [ɑ]/[ke]-power), 2 = incongruent ([i]/[te]-power, [ɑ]/[ke]-precision)]. Another variable in this analysis was Block (1 = vowels, 2 = consonants). This analysis tested whether the congruency effect differs between the blocks. This analysis did not reveal a significant interaction between Block and Congruency [F(1,4307) = 3.52, *p* = 0.061] suggesting that the congruency between Speech unit and Grip type can be observed in both Blocks.

Given that the interaction effect between Speech unit and Response size/Grip type appeared to be smaller in the consonant block of Experiment 1a than in Experiment 1b, we also tested whether this difference is statistically significant. In this analysis, we included the manual data of the consonant blocks of Experiment 1a and 1b into the analysis. The variables that were included in the analyses were Experiment (1 = 1a, 2 = 1b) and Congruency [1 = congruent ([i]/[te]-precision, [ɑ]/[ke]-power), 2 = incongruent ([i]/[te]-power, [ɑ]/[ke]-precision)]. This analysis revealed a significant interaction between Experiment and Congruency [F(1,4288) = 5.52, *p* = 0.019] showing that the congruency effect is indeed significantly larger in Experiment 1b in comparison to Experiment 1a (Experiment 1a: Congruent: M = 537 ms, Incongruent: M = 548 ms, *p* = 0.198, *d*_*z*_ = 0.009; Experiment 1b: Congruent: M = 512 ms, Incongruent: M = 551 ms, *p* < 0.001, *d*_*z*_ = 0.33).

The analysis of intensity showed a significant main effect of Block [F(1,4303) = 6.63, *p* = 0.010]. Intensity was larger in the vowel block (M = 75.3 dB) in comparison to the consonant block (M = 74.5 dB; *d*_*z*_ = 0.17). Considering *f*_*0*_ values, there was a significant main effect of Speech unit [F(1,4143) = 15.97, *p* < 0.001] and a significant interaction between Block and Speech unit [F(1,4143) = 71.66, *p* < 0.001]. In the vowel block, *f*_*0*_ values were higher for [i]-responses (M = 0.27 st) than [ɑ]-responses (M = -0.15 st) (*p* < 0.001, d_z_ = 0.83). In the consonant block, this interaction was not significant ([te]-responses: M = 0.10 st vs. [ke]-responses: M = 0.18 st, *p* = 0.114).

The analysis of *F*_*1*_ values revealed a significant main effect of Speech unit [F(1,4147) = 44.24, *p* < 0.001], Block [F(1,4147) = 17.19, *p* < 0.001], and a significant interaction between Block and Speech unit [F(1,4147) = 3346.95, *p* < 0.001]. In the vowel block, *F*_*1*_ values were higher for [ɑ]-responses (M = 503 Hz) than [i]-responses (M = 302 Hz) (*p* < 0.001, d_z_ = 1.30). In the consonant block, this interaction was not significant ([te]-responses: M = 451 Hz vs. [ke]-responses: M = 451 Hz, *p* = 0.963). The analysis of *F*_*2*_ values revealed a significant main effect of Speech unit [F(1,4248) = 1396.13, *p* < 0.001] and Block [F(1,4248) = 16.01, *p* < 0.001] as well as a significant interaction between Block and Speech unit [F(1,4248) = 76,473.04, *p* < 0.001]. In the vowel block, *F*_*2*_ values were higher for [i]-responses (M = 2680 Hz) than [ɑ]-responses (M = 1104 Hz) (*p* < 0.001, d_z_ = 5.25). In the consonant block, this interaction was not significant ([te]-responses: M = 1821 Hz vs. [ke]-responses: M = 1846 Hz, *p* = 0.253).

The results of Experiment 1 validated the previous finding that when reading a particular speech unit, the speech unit can be systematically linked to facilitated manual responses performed with the small or large response key. However, this key-sound effect was only observed with vowels [i] and [ɑ]. The effect was not observed with keypress responses when participants had to pronounce the consonant [t] or [k]. In contrast, when the responses were performed with the grip devices, the effect was also observed with consonants. In addition, the study revealed that upper/lower responses are facilitated by articulating a high/low vowel, respectively. A similar but less robust effect was also observed with consonants. The alveolar stop consonant [t] was associated with upper responses in the manual RTs, while the dorsal stop consonant [k] was associated with lower responses in the manual and vocal RTs. Finally, the analyses of vocal characteristics did not reveal any effects in which the response location or size would have modulated the values of a vocal characteristic. Only the standard effect of the intrinsic vowel pitch was observed in which the *f*_*0*_ values were higher for the [i] than [ɑ]. In addition, as expected, the *F*_*1*_ values were higher for the [ɑ] vocalizations and the *F*_*2*_ values were higher for the [i] vocalizations.

### Experiments 2a and 2b

Experiment 2 investigates whether the effects observed in Experiment 1 can be replicated when the speech units are not articulated overtly. That is, the participants are required to read the speech units silently while performing the manual responses. It is assumed that if the effects are based on articulatory movements or acoustic consequences of these articulations, they should be greater in the condition of overt articulation observed in Experiment 1.

## Methods

### Participants

Twenty volunteers naïve to the purposes of the experiment participated in Experiment 2 (19–43 years of age; mean age = 29 years; 4 males; 0 left-handed). All participants were native speakers of Finnish and reported normal hearing and normal or corrected-to-normal vision. An appropriate sample size has been determined by Heurley et al. (2023) who presented that a minimum of 14 participants are required to detect an effect size as large as η^2^_p_ = 0.15 in a within-subject design with 95% statistical power (α = 0.05). Two studies have used the same paradigm as the current study to investigate how reading speech units influence responses performed with differently-sized devices. Written informed consent was obtained from all participants. The study was conducted according to the principles expressed in the Declaration of Helsinki. The study was approved by the Ethical Review Board in the Humanities and Social and Behavioral Sciences at the University of Helsinki.

### Stimuli, procedure, and appratus

The apparatuses used in Experiments 2a and 2b and experimental arrangements were similar to those used in Experiments 1a and 1b with the most distinct exception that the microphone was not used in this study as the participants were asked to read the visually presented speech unit silently. The experimenter verified throughout the experiment that the participant did not vocalize the speech unit aloud. Experiments 2a and 2b employed a similar paradigm to that used in the studies by Vainio et al. (2014) and Heurley et al. (2023). At the beginning of each trial, a blank white screen was presented for 2000 ms. Then the speech unit was presented at the center of the screen. The speech unit was a letter < a > (horizontally: 1°; vertically: 1.2°), < i > (0.9°; 1.5°), < y > (1.1°, 1.6°; respectively in centimeters: 1.4 cm/2.1 cm) or < ö > (1°, 1.5°; 1.3 cm/1.9 cm) [or a syllable < ke > (2.1°; 1.5°), < te > (2.2°; 1.5°), < pe > (2.1°, 1.4°; 2.7 cm/1.8 cm) or < ve > (2.3°, 1.1°; 3 cm/1.5 cm) in block 2] presented in the grey color and in lowercase letters with the Consolas font. In Finnish, < a > is pronounced as [ɑ], < i > is pronounced as [i], < e > is pronounced as [e], and < ö > is pronounced as [ø]. After 300 ms the stimulus turned into green (R = 2, G = 157, B = 14) or blue (R = 34, G = 22, B = 250) color. These colored speech units were presented for 800 ms.

In Experiment 2a, the participants responded by pressing one of the two alternative response keys from which one was small and one was large, while in Experiment 2b, they responded by squeezing either the precision or power grip device. Again, a blue or green sticker was placed on each response key/device, and again one response key was closer to the monitor (i.e., the upper key) and one key was closer to the participant (i.e., the lower key). The participants selected the response according to the color of the stimulus. Each participant carried out Experiment 2a before Experiment 2b for the same reason as in Experiment 1. There was a break between Experiment 2a and 2b that lasted for 10 min. During that break, the grip task was instructed to the participants.

Experiments 2a and 2b consisted of two blocks. The participants had a short break between the blocks. In one block, the stimuli consisted of the vowels < i > , < a > , < y > , and < ö > (the vowel block), while the stimuli of the other block consisted of the syllables < te > , < ke > , < pe > , and < ve > (the consonant block). The order of the blocks was randomized between the participants. In addition, the order of the differing stimuli was randomized. In the vowel block, the vowels < y > and < ö > were the catch stimuli, while in the syllable block, the < pe > and < ve > were the catch stimuli. That is, the participants were instructed to withhold their manual response when one of these catch stimuli was presented. These catch trials were included in the design in order to encourage the participants to read the speech units instead of solely focusing on the color. When the participant responded incorrectly (i.e., they responded in the catch trial or they responded with the wrong key/device), a red”error” text was displayed at the center of the screen for 800 ms. In both experiments, 208 trials presented a target stimulus that called for a response, and 104 trials presented a catch stimulus. In total, Experiments 2a and 2b lasted around 15 min and consisted of 208 target trials [26 × 2 (block) × 2 (speech unit) × 2 (response)].

In Experiment 2a, there were four different response settings: 1a) the large-green key upper; the small-blue key lower; 2a) the large-blue key upper; the small-green key lower; 3a) the small-green key upper; the large-blue key lower; 4a) the small-blue key upper; the large-green key lower. Twenty participants were evenly allocated to each of these response settings. In Experiment 2b, there were two different response settings: 1b) the precision grip-blue; the power grip-green; 2b) the precision grip-green; the power grip-blue. Half of the participants performed the task in response setting number 1b.

### Statistical analyses

Prior to analyzing RTs, the catch trials were removed from the data. The participants responded in only 1.7% of the catch trials. In addition, the errors [i.e., the participant produced a wrong manual response (Experiment 2a: 2.6%; Experiment 2b: 2.4%), and the conditions in which a participant did not produce any response (Experiment 2a: 1.3%; Experiment 2b: 0.8%) were removed from the data. None of the participants produced reaction times below 150 ms.

Separate analyses were executed for Experiments 2a and 2b. The statistical significance of observed differences was tested using the generalized linear mixed model (GLMM) analysis framework. The GLMM analyses treated the independent variables of Block (1 = vowels, 2 = syllables), Speech unit (1 = [i]/[te], 2 = [ɑ]/[ke]), and Response size/location (1 = small/upper, 2 = large/lower) (or Grip type as a Response factor in Experiment 2b) as fixed within factors. In addition, there was a random intercept of Subject with a random slope of Block, Speech unit, and Response. The residual distributions were significantly positively skewed, so a gamma distribution assumption (log link function) was implemented. All pairwise comparisons were carried out using Bonferroni correction for multiple comparisons. The analyses were carried out using the SPSS statistics software package (version 28).

## Results and discussion

### Experiment 2a (keypress)

**Fig. 5:**
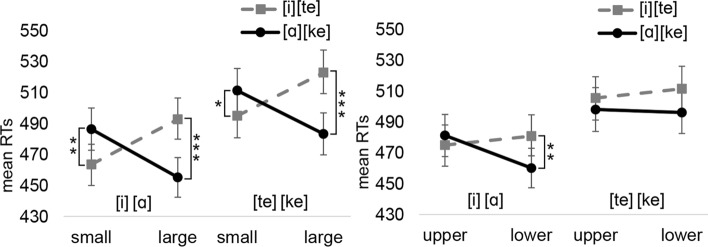
The mean manual reaction times for Experiment 2a as a function of the speech unit and response size (left) and the speech unit and vertical response location (right). Error bars depict the standard error of the mean. Asterisks indicate statistically significant differences (*p < .05, **p < .01, ***p < .001)

*The interaction between the speech unit and the small/large response*: The analysis of reaction times revealed the main effect of Block [F(1,3986) = 22.01, *p* < 0.001] and interaction between Speech unit and Response size [F(1,3986) = 63.95, *p* < 0.001]. The three-way interaction was not significant (*p* = 0.591). The interaction between Speech unit and Response size was significant in both blocks (Block 1: small-[i]: M = 464 ms vs. small-[ɑ]: M = 486 ms, *p* = 0.003, *d*_*z*_ = 0.27; large-[i]: M = 493 ms vs. large-[ɑ]: M = 455 ms, *p* < 0.001, *d*_*z*_ = 0.45; Block 2: small-[te]: M = 495 ms vs. small-[ke]: M = 511 ms, *p* = 0.049, *d*_*z*_ = 0.18; large-[te]: M = 523 ms vs. large-[ke]: M = 483 ms, *p* < 0.001, *d*_*z*_ = 0.43). The interactions between Speech units and responses are presented in Fig. 5 (left).

We also tested whether the congruency effect between Speech unit and Response size is significantly different between the vowel and consonant block and whether the location of the response key influences the congruency effect between a speech unit and the small/large size of the response. For that purpose, Speech unit and Response size were transformed into a single independent variable of Congruency [1 = congruent ([i]/[te]-small, [ɑ]/[ke]-large), 2 = incongruent ([i]/[te]-large, [ɑ]/[ke]-small)]. Other independent variables in this analysis were Response location (1 = upper, 2 = lower) and Block (1 = vowels, 2 = consonants). This analysis did not reveal a significant difference in the congruency effect between the blocks (Block*Congruency: *p* = 0.625). In addition, the response location did not influence the congruency effect (Response location*Congruency: *p* = 0.401; Response location*Block*Congruency: *p* = 0.191).

*The interaction between the speech unit and the upper/lower response*: The analysis showed a significant main effect of Block [F(1,3986) = 21.92, *p* < 0.001] and interaction between Speech unit and Response location [F(1,3986) = 5.88, *p* = 0.015]. The interaction between Block, Speech unit, and Response location was not significant (*p* = 0.170). In the lower location, responses were significantly faster when the vowel was [ɑ] (M = 460 ms) rather than [i] (M = 481 ms) (*p* = 0.007, *d*_*z*_ = 0.23). The other interactions were not significant (Block 1–upper: [i] M = 475 ms, [ɑ] M = 481 ms, p = 0.425; Block 2–upper: [te] M = 505 ms, [ke] M = 498 ms, *p* = 0.370; lower: [te] M = 511 ms, [ke] M = 496 ms, *p* = 0.064). Figure 5 (right) presents the interactions between Speech units and Response locations.

We also analyzed the data so that Speech unit and Response location were transformed into a single variable of Congruency [1 = congruent ([i]/[te]-up, [ɑ]/[ke]-down), 2 = incongruent ([i]/[te]-down, [ɑ]/[ke]-up)]. Other variables in this analysis were Response size (1 = small, 2 = large) and Block (1 = vowels, 2 = consonants). This analysis showed that the congruency effect was not significantly different between blocks (Block*Congruency: *p* = 0.190). In addition, Response size did not significantly influence the congruency effect (Response size*Congruency: *p* = 0.399; Response size*Block*Congruency: *p* = 0.445).

### Experiment 2b (grip squeeze)

**Fig. 6:**
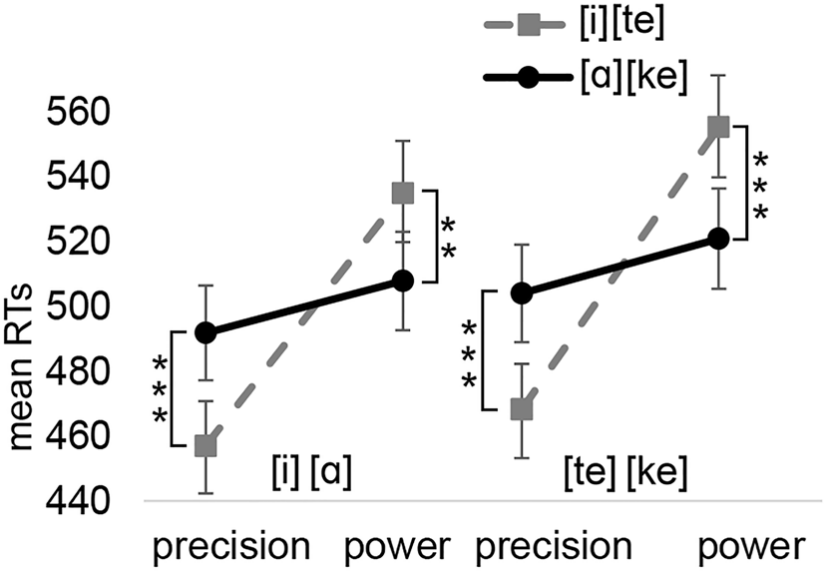
The mean manual reaction times for Experiment 2b as a function of the speech unit and grip type. Error bars depict the standard error of the mean. Asterisks indicate statistically significant differences (*p < .05, **p < .01, ***p < .001)

*The interaction between the speech unit and the precision/power response*: The analysis showed a significant main effect of Grip type [F(1,4019) = 25.85, *p* < 0.001] (precision: M = 480 ms vs. power: M = 529 ms, *d*_*z*_ = 0.52). The interaction between Speech unit and Grip type was also significant [F(1,4019) = 70.86, *p* < 0.001]. However, the three-way interaction was not significant (*p* = 0.700). The interaction between Speech unit and Response size was significant for both speech units in the vowel and consonant blocks (precision-[i]: M = 457 ms vs. precision-[ɑ]: M = 492 ms, *p* < 0.001, *d*_*z*_ = 0.35; power-[i]: M = 535 ms vs. power-[ɑ]: M = 508 ms, *p* = 0.006, *d*_*z*_ = 0.25; precision-[te]: M = 468 ms vs. precision-[ke]: M = 504 ms, *p *< 0.001, *d*_*z*_ = 0.35; power-[te]: M = 555 ms vs. power-[ke]: M = 521 ms, *p* < 0.001, *d*_*z*_ = 0.30). Figure 6 presents these interactions.

Contrary to the results of Experiment 1, the results of Experiment 2 revealed that when the speech units are red silently, the interaction between manual responses and a particular speech unit is similar with vowels and consonants regardless of whether the manual responses are performed with the small/large response keys or the precision/power grips. Furthermore, the effect concerning upper and lower responses observed in Experiment 1a was not properly replicated in Experiment 2a suggesting that overt articulation boosts this effect (Table 1).

**Table 1:** The interaction effects observed in vocal and/or manual reaction times between Speech unit (SU) (vowels: [i] and [ɑ]; consonants: [t] and [k]) and Response (Response size: small and large; Response location: upper and lower; Grip type: precision and power) in Experiments 1a, 1b, 2a, and 2b

Experiment	Vocal RTs	Manual RTs
Exp1a: small/large keypress + vocal	Vowels: SU * Resp. size for [i] and [ɑ] Consonants: no interactions	Vowels: SU * Resp. size for [i] and [ɑ] Consonants: no interactions
Exp1a: upper/lower keypress + vocal	Vowels: SU * Resp. location for [i] and [ɑ] Consonants: SU * Resp. location for [k]	Vowels: SU * Resp. location for [i] and [ɑ] Consonants: SU * Resp. location for [t] and [k]
Exp1b: prec/pow grasp + vocal	Vowels: SU * Grip type for [i] Consonants: SU * Grip type for [t] and [k]	Vowels: SU * Grip type for [i] and [ɑ] Consonants: SU * Grip type for [t] and [k]
Exp2a: small/large keypress	n.a	Vowels: SU * Resp. location for [i] and [ɑ] Consonants: SU * Resp. location for [t] and [k]
Exp2a: upper/lower keypress	n.a	Vowels: SU * Resp. location for [ɑ] Consonants: no interactions
Exp2b: prec/pow grasp	n.a	Vowels: SU * Grip type for [i] and [ɑ] Consonants: SU * Grip type for [t] and [k]

## General discussion

The study validated the previously presented observation (Heurley et al. 2023) that reading particular speech units facilitates manual responding with small and large keypresses when there is an overlap between a sound-symbolic connotation of a speech unit and the size of the manual target. This effect occurs even though the size information is an entirely task-irrelevant aspect in the speech unit and the response. We agree to some degree with Heurley et al.’s view that this key-sound effect might reflect magnitude-related compatibility between the response code and the speech unit. Moreover, we propose that reading of a particular speech unit conveys a magnitude-related sound-symbolic connotation (e.g., [i]–small), either due to acoustic or articulatory elements associated with it, as presented in the Introduction. As assumed by accounts of embodied cognition (Barsalou 2008), representing this kind of abstract conceptual information recruits sensory, emotional, and motor processes. As a consequence, the responses that match the sound-symbolic connotation of the speech unit (e.g., [i]–small manual target) are produced faster than those that do not match.

The study also replicated the grip-sound effect (Vainio et al. 2013). However, in light of the present findings, it cannot be stated conclusively whether the grip-sound effect is solely based on the interaction between the grip type and the sound-symbolic connotation of the speech unit, or whether the effect is more based on similar processes as the key-sound effect in which the size of the response target is a more determinative factor of the effect than the grip type. It has been previously shown that the perceptual processing of grasp information systematically influences the vocalization of vowels that sound-symbolically match this information. Firstly, the viewed precision grip facilitates vocalizing the vowel [i], while the viewed power grip facilitates vocalizing the vowels [ɑ] and [o] (Vainio et al. 2017). Secondly, viewing a picture of an object affording either the precision grip (e.g., a pin) or the power grip (e.g., a banana) facilitates vocalizing the vowels [i] and [ɑ], respectively (Vainio et al. 2019). Moreover, in one of the previous studies (Vainio et al. 2023a), the participants were presented with a pseudoword that consisted of vowels and consonants that were hypothesized to be associated with the precision grip (e.g., *hitesiti*) or power grip (e.g., *mangakha*). They were simultaneously presented with two animated hand action videos: one that manipulated an object with the precision grip, and one that manipulated an object with the power grip. The experimental task was to select which one of the action videos provides a better match to the pseudoword. It was found that pseudowords such as *hitesiti* were more often linked to precise manipulation, while pseudowords such as *mangakha* were more often linked to power manipulation. These three studies provide evidence for the view that a particular grip type is implicitly associated with particular speech units. In light of this evidence, given that vocalization is consistently influenced by processing grasp information, it appears possible that the grip-sound effect, which also connects grasp information to vocalization in the overt vocalization tasks, is at least to some extent based on the interaction between the grip type and the sound-symbolic connotation of the speech unit.

The study also showed that the grip-sound effect is observed more robustly with consonants in comparison to the key-sound effect. In particular, in Experiment 1a, which required overt articulation of the speech units, the key-sound effect was missing with the consonants [t] and [k]. In contrast, in the corresponding vocalization task of Experiment 1b, the grip-sound effect was robust. This finding supports the view (Vainio et al. 2017, 2019) that the grip-sound effect linked to producing the consonants [t] and [k] might be grounded in a concrete mapping between grip type and articulatory gesture that requires an overt formation of a particular articulatory shape as well as a grasp execution. In contrast, when high-front and low-back vowels are articulated, producing these vowels might provide relatively univocal proprioceptive and visual feedback about the magnitude of the opening of the lips and oral cavity as well as pitch-related acoustic information that can also link these vowels to particular magnitudes. As a consequence, the sound-symbolic magnitude of these vowels can be conceptually represented in a relatively abstract manner. Therefore, these kinds of abstract magnitude-concept representations can be in turn implicitly mapped to similarly abstract small/large response representations resulting in the key-sound effect.

The key-sound effect was, however, observed with the consonants [t] and [k] in Experiment 2a when the participants had to read the speech units silently without overt articulation. An interesting question is: why the key-sound effect is observed with consonants when reading the consonants covertly but is not observed when reading them overtly? As stated above, it is possible that when the task requires overt articulation of speech units, as in Experiment 1, the concrete action processes (e.g., articulating a particular consonant and producing a particular grasp) are emphasized in mapping the manual response to the produced speech unit. In contrast, when the task does not require actual articulation of the speech unit, as in Experiment 2, more abstract associative processes might dominate the mapping between the response and speech unit resulting in the key-sound effect also with the consonants. Similar abstract associative processes might be in operation in traditional sound-magnitude symbolism phenomena in which, for example, [t] is intuitively linked to small magnitudes and [k] is linked to larger magnitudes (Newman 1933; Winter and Perlman 2021).

The manual responses were not observed to modulate spectral components of intensity, *f*_*0*_, *F*_*1*_, or *F*_*2*_, and the interaction effects observed in reaction times were not reflected in the values of these spectral components. The only effects that were observed in spectral components were associated with differences in articulation of specific vowels. As reported in numerous observations (e.g., Fant 1960; Whalen and Levitt 1995), the intrinsic vowel pitch was higher for [i] than [ɑ] and *F*_*1*_ was higher for [ɑ] whereas *F*_*2*_ was higher for [i]. The lack of effects critical to the congruency effects observed in reaction times can be taken to suggest that these congruency effects might reflect cognitive overlaps in processes that operate for selecting manual (e.g., precision vs. power grip) and vocal (e.g., consonant [t] vs. [k]) responses and that fully operate before the onset of the actual response.

### The interaction between speech units and upper-lower responses

Importantly, the study also showed that the upper responses are associated with the vowel [i] and consonant [t], while lower responses are associated with the vowel [ɑ] and constant [k]. This was particularly observed in Experiment 1a. The main limitation of this finding is linked to using the vowel [ɑ]. It has to be emphasized that these speech units were used because the experimental setup for investigating this location-sound effect was subordinate to the experimental setup for investigating the grip-sound and key-sound effects. Therefore, the stimulus (i.e., the visually presented vowels and consonants) that were employed to study the location-sound effect were primarily selected for investigating the grip-sound and key-sound effects. However, the vowel [ɑ] plays a key role in the Finnish words [ɑlɑs] (down) and [ɑlempi] (lower). As such, it is possible that the occurrence of this sound in the words that refer to the concepts *down* and *lower* might have been the driving force in the interaction effect observed between the [ɑ] and lower responses. Nevertheless, similar limitations are not linked to the speech units of [t], and [k]. Hence, at least from those parts, the location-sound effect can be considered to be a genuine sound-response interaction effect. Therefore, it can be argued that the present study replicated the location-sound effect, previously observed in the interaction between vocalized vowels and vertical movement of visual stimulus (Vainio et al. 2023b) in the context of producing particular consonants, by replacing the vertical positions of visual stimuli with the vertical positions of responses. The question of whether a similar effect can be also observed in vowel production warrants further investigation.

Another shortcoming of this study was that although we were interested in whether processing particular speech units for responses systematically influences responding with the upper and lower response keys, the response keys were not, in fact, arranged within a vertical axis. Instead, the responses were performed in the sagittal axis in the same way as in the study of Rusconi et al. (2006). Therefore, even though the participants were instructed to process these keys as an upper and lower rather than further and nearer, there is a possibility that the results of this study as well as the SMARC effect (Rusconi et al. 2006) are based on processing the response keys as a further and nearer. In that case, instead of replicating the interaction effect between vocalized speech units and vertical movement of visual stimulus (Vainio et al. 2023b) in the context of manual responses, the study might have replicated the sound-distance effect in which particular speech sounds have been observed to be linked to particular distances (Woodworth 1991; Rabaglia et al. 2016; Vainio 2021). However, given that the sound-distance effect links front-high vowels to the concept of near and back-low vowels to the concept of far, if the present effect were based on processing distance information for performing responses, the vowel [i] should have been associated with nearer (i.e., lower) responses and the vowel [ɑ] should have been associated with further (i.e., upper) responses. We observed the opposite effect to that suggesting that the present effect is indeed associated with processing responses in the vertical axis.

Given that the corresponding location-sound effect can be observed with the visual stimuli that move up or down as well as with the upper and lower manual responses, we propose that the effect reflects the embodied grounding of the spatial concepts *up* and *down* in sensory, emotional, and motor processes. This view holds that reading, for example, the speech units of [i] or [t] results in representing these speech units at the conceptual level in a sound-symbolic manner so that they are grounded in the abstract spatial representation of the *up* concept. This occurs either because the alveolar stop consonant [t] as well as the vowel [i] have higher spectral components than the dorsal stop consonant [k] or the vowel [ɑ] (Chodroff and Wilson 2014; Whalen and Levitt 1995). Alternatively, [i] and [t] could be a better match for the upper location than [ɑ] and [k] because when articulating [i] and [t], the tongue tip moves upwards, while when articulating [ɑ] and [k], it moves downwards. Nevertheless, the fact that the location-sound effect was nearly exclusively observed when the participants were required to overtly produce the speech units (Experiment 1a) in comparison to the condition in which they had to read the speech units silently (Experiment 2a) suggests that the effect is largely based on articulation processes or the acoustic consequences of these processes. One might argue that because speech motor plans are most likely also activated when reading quietly (Galantucci et al. 2006), the acoustic consequence might be the stronger candidate for the explanation.

In conclusion, the study investigated two interaction effects: one that shows the interaction between the particular speech units and small/precision and large/power responses, and another that shows the interaction between the particular speech units and upper and lower responses. To some extent, both of these effects were assumed to reflect representing particular speech units at the conceptual level in a sound-symbolic manner. For instance, reading the vowel [i] results in representing this vowel in relation to the concepts of *small* and *up* because the intrinsic vowel pitch of this vowel is particularly high and/or because this vowel is produced by raising and fronting the tongue. Given that these conceptual representations might be partially grounded in the motor processes in addition to perceptual processes (Barsalou 2008), reading this vowel can result in implicitly mapping it to the manual responses, which in turn can be observed in facilitated responses performed with the upper and smaller response keys as well as with the precision grip. Finally, the key-sound effect was not observed with the consonants [t] and [k] when the task required overt articulation of these consonants, while the grip-sound effect was observed robustly in the same conditions. This was taken to indicate that the grip-sound effect linked to producing the consonants [t] and [k] might be grounded in a relatively concrete mapping between grip type and articulatory gesture that require overt articulation of the consonant and actual grasp execution.

## Data Availability

The data is available in the following repository https://osf.io/u3pxe/
